# Meteorological Table

**Published:** 1811-09

**Authors:** 


					[ 263 ]
METEOROLOGICAL TABLE.
From July 2S, to August ^0. tt . .
I Therm. | Barom.l Hygrom
A-tmos. Variation,
?;28!66 SI 7r30 299i
i29';68 78 681299 30
[30:64 69 66301 ?
|3161 67 651301 30
1(60 72 67130 ?
2:63 75 64i299 ?'
3(65 75 65|29'7
0
4"j59 68 61
560 67 61
6 60 63 59
|297
129 6
297
7 57 69 64 297
8 58 62 56 295
9 55 60 56 29s
' 7 53 60 53|296
51 60 54
52 63 ."8
60 70 63 30
1299
302
30'
|l4l59 67 57
'56 67 56
|302
r29 ?3
? 3
12 ?
. 4
58 65 58 30
55 70 62
59 73 63
*9 63 67 60
20 60 68 56,
21 59 67 ?
22,62 67 64
60 67 63 30 293
23,
124-161 68 64,
25(63 66 60
26 56 69 61
301
302
|293
29 s
i301
1301
14 4 ..
15 26 401SW
40 38 4|NW..
16 20 21 IN .. w
24 19 22 NW..
17 15N ..
7 9 NE . .
10 181 NW ..
?|w..
15 NW
30 !?
7
30
30
29 s ?'1
295 ?1
vri ?<
19
11
22
17
15
50 ?
- 10
122 ?
122 36
127 ?
10
24 14
? 15 ?
22 NW.
15IW . .
5'W . .
5|E. SE.
10 22 20; W .. SW .
22 SW . ?
NW.
? 10
33 27!W.
122 |SW
30 19 22 SW
?'sw
!w.
125 33 25
8 27 15 ?
R u 1U!tnt"y li"n from July 28 to August 26, 1 inch and -j^s>
th. Thunder in the middle of the day, with heavy rain.
r Thunder with rain at nine A. M. Frequent distant thunder in the
Ol'encon.
int^0t^ thermometer an<i barometer have had an extensive range in this
^1 e.fVal* On the 28th of July the thermometer at 2 o'clock P.M. stood at
0n Ae 9th, lOth9 and 11th of August it stood at (SO, at the same hour,
sunl- mercury in the barometer has risen to 303 on the 14th of August, and
that" t-? 0n t'ie anc* ^ t'lat mont^* ^ may be observed
*her ""ain \'13S regularLv followed the depression of the mercury, and fair wea-
inj- lts Ovation, The hygrometer has been remarkably fluctuating, and has
?f th* approaches of rain or fair weather very faithfully. The humidity
foeen e mornings and evenings has been considerable, when the air at noon has
gu ^ery dry. There has been one exception to this. On the 26th of Au-
o notwithstanding the atmosphere was clear, and apparently dry, the hy-
ln^^ate^ mucb humidity; but on the cloudy evening of that day
enmg rain, it rose to eight degrees of drv.
rinces Street, Cavendish Square.

				

